# Supplementary figures and images for: How distinct sources of nuisance variability in natural images and scenes limit human stereopsis
Source: PLoS Comput Biol. 2025 Apr 15;21(4):e1012945. doi: 10.1371/journal.pcbi.1012945 (PMC12080933; doi:10.1371/journal.pcbi.1012945)

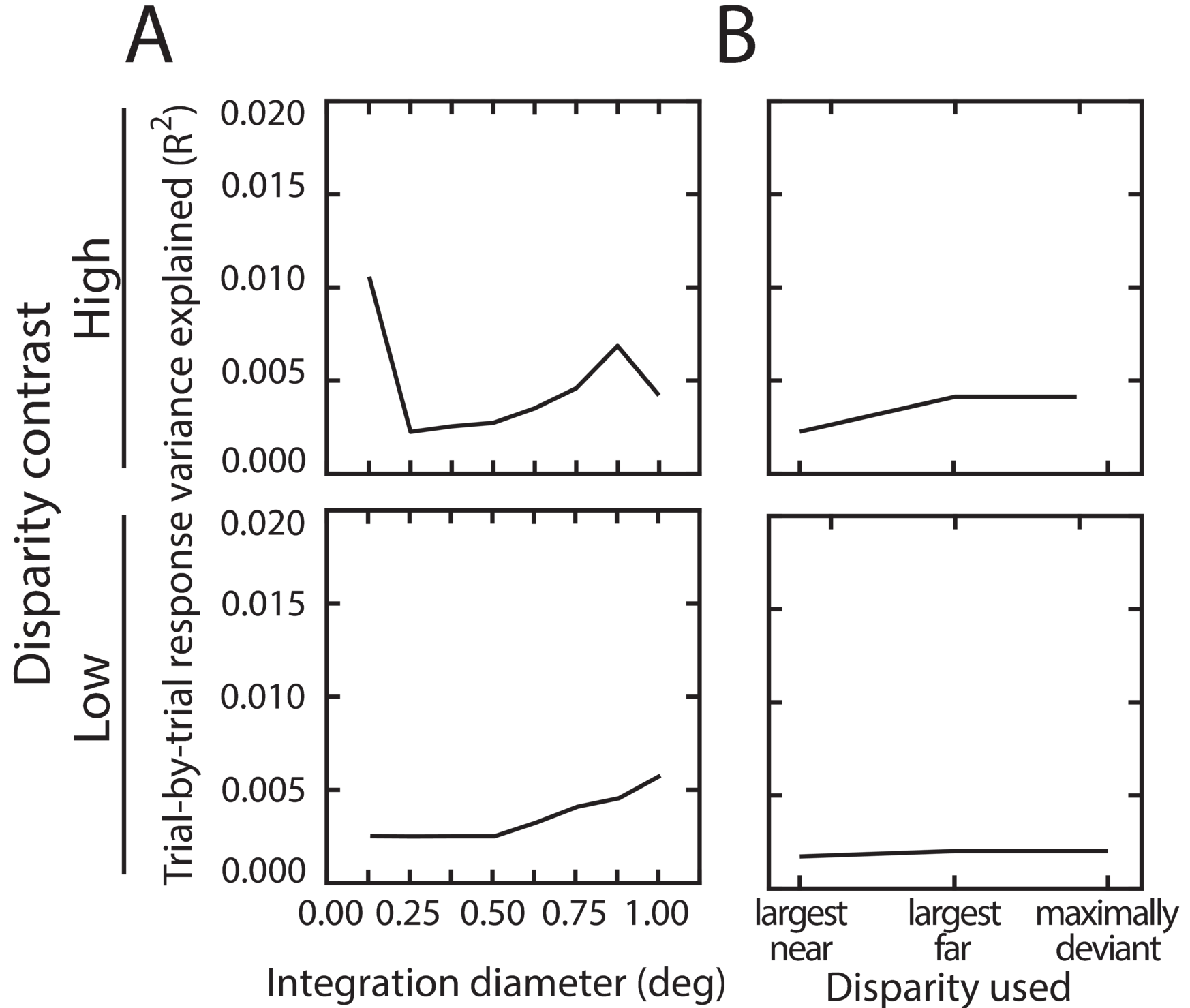

Supplement: S1 Fig — Logistic regression was used to assess whether a number of different strategies could account for the variability in trial-by-trial responses in the high (top row) and/or low (bottom row) disparity-contrast conditions. (A) Variance accounted for by strategies that assume a fixed spatial-integration area as a function of integration diameter, where each disparity estimate is computed as the mean disparity within the integration area of each patch. Note that the largest integration diameter is equal to the area of the entire patch, and the smallest integration area was equal to the central region which had the same disparity value at each pixel up to a tight tolerance. In the former case, the disparity estimate equals the mean disparity of the patch. In the latter case, the disparity estimate equals the disparity of the central, target pixel. (B) Variance accounted for by strategies that assumed that the decision variable was determined by the largest near disparity (nearest), largest far disparity (farthest), and maximally deviant disparity (max) of each patch. (TIF) [file pcbi.1012945.s001.tiff]

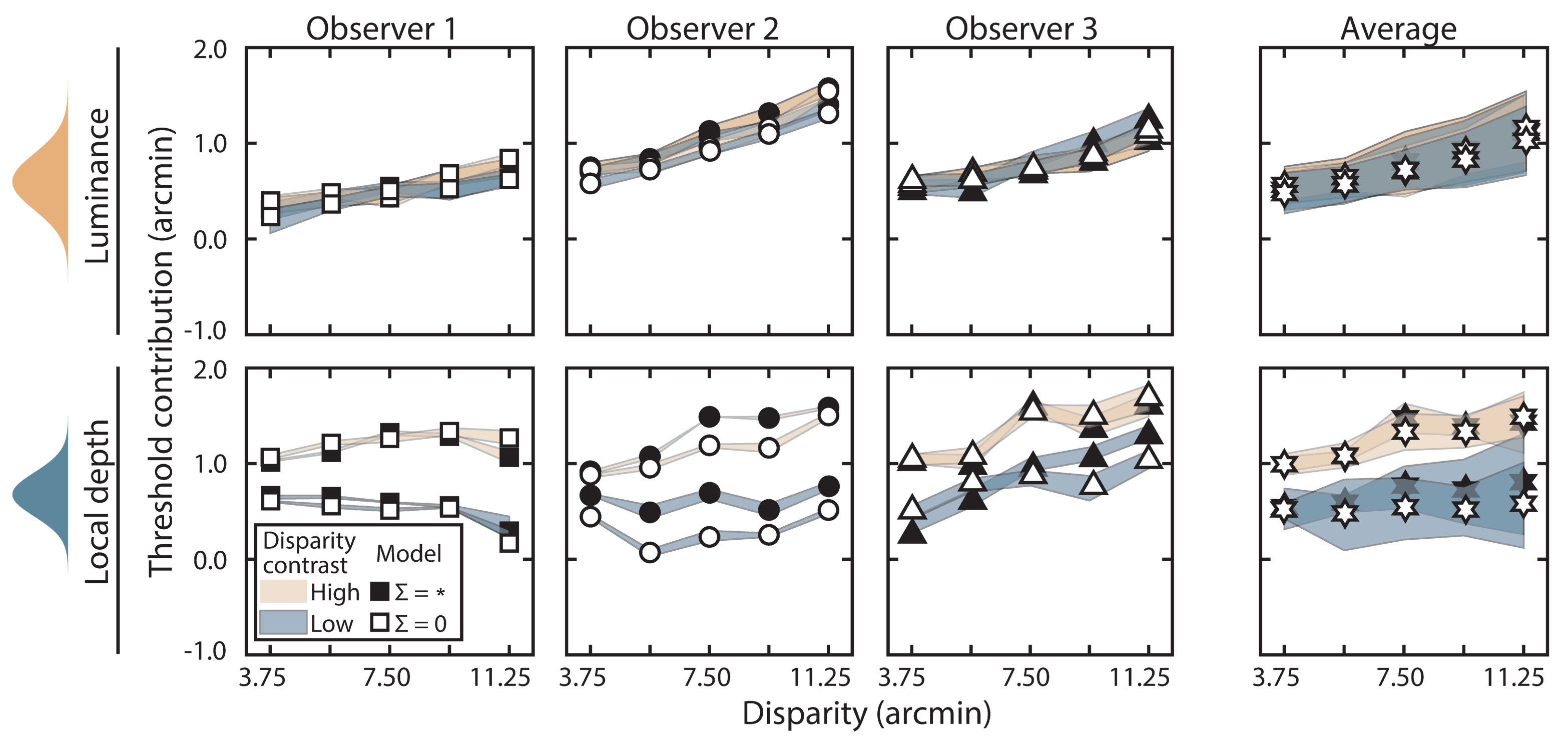

Supplement: S2 Fig — For reference, threshold-contributions fit without the zero-covariance constraint are also shown (black-filled symbols). Constraining the covariance has little effect for Observer 1, Observer 3, and the Average Observer. In Observer 2, the magnitudes of the threshold contributions are systematically reduced, but the patterns are unaffected. In cases where black-filled symbols are not visible, they are plotted directly behind white-filled symbols. Contribution of luminance pattern variability (top) and variability in local-depth structure (bottom) to threshold as a function of disparity pedestal at different disparity-contrast levels (shades), for each observer and the observer average. For individual observers, shaded regions indicate 68% confidence intervals for each condition, generated from 10,000 bootstrapped samples. For the observer average (last column), shaded regions indicate across-observer standard-deviations. (TIF) [file pcbi.1012945.s002.tiff]
