# Supplementary material for: How distinct sources of nuisance variability in natural images and scenes limit human stereopsis
Source: PLoS Comput Biol. 2025 Apr 15;21(4):e1012945. doi: 10.1371/journal.pcbi.1012945 (PMC12080933; doi:10.1371/journal.pcbi.1012945)
Supplement: Attachment — Submitted filename: WhiteBurge_PLoSCB_2025rsp2rvw.docx [file pcbi.1012945.s003.docx]

Dear Editors:

We thank you and the reviewers for the thoughtful and constructive reviews. We have submitted a revised manuscript, which we believe is much improved thanks to these reviews. Below you will find point-by-point responses to them. We hope you and the reviewers find the revision suitable for publication in PLoS Computational Biology.

Best,

David White

Johannes Burge

***

**Note that line numbers refers to the revised manuscript with tracked changes.**

*Reviewer #1: Review of "How distinct sources of nuisance variability in natural images and scenes limit human stereopsis" Reviewer: Michael Landy

This is a really interesting and well-done piece of work that comes to the conclusion that a surprising amount of variability underlying disparity judgments (of patches of natural images) is driven by stimulus variability rather than internal noise, and that the stimulus variability comes from two effectively independent sources (disparity pattern variability and luminance pattern variability). The data are convincing, the methods are perhaps partially drawn from previous work, but pushed to a new level in terms of the conclusions one can draw. All around impressive. But, the Methods section was quite a slog and could be made a bit easier to parse.*

Thank you for these remarks. *Comments, bigger and trivial (page/para/line):

6/3: I would hope the authors can share more than just data (e.g., analysis scripts, etc.).*

As requested, we have uploaded analysis scripts, to [https://github.com/burgelab/WhiteBurge202](https://github.com/BurgeLab/WhiteBurge2025)5-Code. We have also published a MATLAB package that runs the decision variable correlation fitting routines: [https://github.com/burgelab/PsyAgreeMat](https://github.com/BurgeLab/PsyAgreeMat).

*7 et seq.: I'm still a little confused about the stimuli. The surround is meant to be at zero disparity with the image behind (and the "center" of the image at an uncrossed disparity that is one of the experimental parameters that is varied). The raised-cosine is kind of like a blurry or frosted window through which the scene is viewed. What is the disparity of the window's edge?*

The disparity of the fuzzy window was zero arcmin, so it was stereoscopically indicated to be at the distance of the display. The reviewer is correct that the stimulus was stereoscopically specified to be behind the fuzzy window.

*I assumed it was at zero disparity, in which case more should be seen of the right eye's view at the left edge of the stimulus and vice versa. But, some of the text seems to indicate that a circular patch is cut from both eyes' views centered on the central corresponding point.*

If a stimulus had zero disparity, then the left- and right-eye images would be ‘cut’ from the central corresponding point. If a stimulus had uncrossed disparity, a virtual pair of eyes would be fixated ‘in front’ of the surface along the cyclopean line of sight to the depicted surface, such that the corresponding point would have the appropriate disparity.

*Which is it, and please clarify that in the manuscript. If it's the latter, then I'm not sure how to think about the scene (mid-gray in front, but what happens at the edges to vignette the scene that's behind it?). I was worried that, e.g., if the standing disparity were 1 deg (and the former version of vignetting was used), there would be NO correspondence.*

It is correct that if the standing disparity were 1 deg, there would be no visible corresponding points “through” the fuzzy window. But the largest uncrossed disparity that was presented in the experiments was 15.0 arcmin.

*Obviously, that's not what you meant. So, clarify the situation early, please. One confusion is in the definition of v_0, the vergence required to fixate the two center pixels. Maybe I missed it while reading, but I still wasn't sure when I got to this line that the center pixels corresponded.*

As noted, the window is always at zero disparity. The disparity of the central pixel of the standard stimulus reflects the disparity condition of the experiment. We have updated the manuscript to clarify all this at line 118. It reads:

“Image patches were presented dichoptically and subtended 1° of visual angle. The center pixel of the stereoscopically-specified scene patch was located straight-ahead along the observer's cyclopean line of sight and had uncrossed disparity with respect to the display. The patch was spatially windowed by a raised-cosine function---a Hann window function---having zero disparity with respect to the display. When viewed binocularly, the patch of scene appeared in depth behind a fuzzy aperture; the aperture appeared in the depth-plane of the display. When viewed monocularly, the patch appeared to fade into the mean luminance surround (see Fig 2)

And later at line 148 to help clarify the definiton of vergence demand v_0:

“Note that that $\mathbf v_0$ is identical for all stimuli having the same nominal disparity, but that the differences in vergence demand $\mathbf{v(x)}$ - $\mathbf v_0$ (i.e. the pattern of relative disparity) is unique to each stimulus.”

*Eq. 2: Does this pool both images? Using the same scaling?*

Yes. Equation 2 has been updated to reflect this.

*8/2: Here is where you say the patches are centered on corresponding points, at last.*

As noted, the patches were initially centered on corresponding points, but then uncrossed disparity was added through cropping. We found the use of “centered on corresponding points” to be too ambiguous, and have now opted for “straight-ahead along the observer's cyclopean line of sight.” This point is now made several paragraphs earlier in the manuscript (see above responses).

*8/3/5: "central region": You should give its diameter here.*

It was written further down. We’ve updated the manuscript to include it here.

*8/3/7: This would be better rephrased by giving the purpose first: "We are interested in the effect of disparity contrast on performance, so we chose patches whose disparity contrast fell either into a "low" range (details) or a "high" range (details), and we report performance below for each group of images separately."*

Good suggestion. Changed as requested at line 167, which now reads:

“Because we were interested in the effect of disparity contrast on performance, we sampled patches whose disparity contrast, when viewed in the experimental rig, fell into a "low" range (0.025-0.117 arcmin) or a "high" range (0.393-1.375 arcmin).”

*8/4/3: I'm not sure it's cool to cite something as "n.d." with no source or means for a reader to find the citation.*

This was a document compilation issue. Fixed. (Held & Banks, 2008).

*10/2: I'd like a figure panel that shows the stimulus in situ with the background and "reticle" (whatever that is!). What's an "ordinal" direction? (New use of that word for me...)*

We’ve inserted a new figure (Fig 2 in the revision) which illustrates the experimental task, and shows the stimuli as they appeared in the experiment.

Reticle is a term for things like crosshairs. Ordinal directons = directions of main digonals. The fact that these words were unfamiliar led us to change our terminology in the updated mansucript.

*11/5/2-3: 5x5x2 does not equal 10. Later, comparison disparity is NOT treated as a condition (but rather as an independent variable).*

This was a typo. Apologies. It now indicates that 5 x 2 = 10 conditions.

*Eq. 4: d' is usual separation/spread, so why is it over variance here??? S/N is usually in energy terms, so separation^2/variance. This equation is neither.*

Fixed. Was a typo.

*13/1/5: Section 4.6 -> Eq. 6*

Fixed.

*Eq. 11: This tripped me up, because I stupidly looked at the equation and thought "r^2 is the proportion of variance accounted for, not r!" I was wrong, because this is not linear regression but the correlation between two variables with shared and exclusive variance, and that comes out differently. But, for readers who might be similarly tripped up by leaping to a false analogy, you might warn them. Maybe it's just me, though ;^/*

We’ve added a note to the reader in order to hedge against this at line 323. It reads:

“(Note that decision variable correlation \cref{eq:rho} should not be confused with variance accounted for ($R^2$), a statistic that is often computed in regression analyses.)”

*Page 16, et seq.: This feels a bit like teaching rather than developing the math needed here. You are going to do a case that is symmetric and with a criterion at zero, but you develop the math for a general case with two different means (for the two passes) and nonzero criteria. Why bother? Once you assume zero and, in particular, equal criteria, and equal means for the two passes, the picture is symmetric (so that +- and -+ come out the same). It would be nice to save some verbiage by going with what is actually happening here from the get go. This will simplify Eq. 17, for example (using symmetry).*

You are correct that these equations simplify for the relevant analyses of the paper. We’ve added to the document so that it includes this simplification. We also failed to mention that we first used the general non-symmetric form to help verify the integrity of the results. The document now includes the following at line 368:

“The decision-variable correlation analyses presented in this work were computed under the assumption that the means were equivalent and criteria were zero. Under these assumptions, the two likelihoods of response disagreements are equal ($\mathcal{L}_2^{-+}=\mathcal{L}_2^{+-}$), thus simplifying the computation of \cref{eq:lls2D}). However, we first verified that these assumptions were reasonable for the current dataset.”

*21/1/3: "depthnzz"*

Fixed.

*22/2/3: inconsistent WITH the expansion*

Fixed.

*22/2/11-12: twice "subscripts [to] denote" (delete "to")*

Fixed.

*24/2/1: with-observer -> within-observer*

Fixed.

*25/2/4: asses -> assess ;^)*

Fixed. No donkeys were harmed in the writing of this article.

*25/4/3: Here's where you list 10 conditions the right way*

Fixed.

*Figure 2A: The Pass 2 timeline is missing the stimulus in the 2nd interval.*

Fixed.

*32/1/3: parallel to ONE another*

Fixed.

*32/2/1: nobody -> no one*

Fixed.

*f33/3/6: You should probably show the data (as a function of pooling radius)*

The results do not merit a plot in the main text; all R^2 values (variance in trial-by-trial responses explained) were all less than 0.01. However, in the manuscript, we now indicate how small these values were: “(all R^2 < 0.01).” For the sake of transparency, the requested data plot is included as a supplemental figure (Fig S1).

*Eqs. 48 and 49: What's with all that space in the parentheses in the denominators?*

We’ve removed them in equations 45 and 46 as requested. We’ve reatained them where we believe it eases visual comparison of the equations relating the flattened and non-flattened conditions (equation 22).

*41/3/4: These findings should also probably be shown in a figure.*

In the main text, we’ve added a new figure (Fig 12 in the revision) showing the estimates of the local-depth- and luminance-driven uncertainty and of internal noise are essentially unchanged when estimated with the model that forces the covariance between L and B to be zero.

We have also added a supplementary figure (Fig S2) that overlays results using both models and explicitly shows the estimated covariance values.

*Fig. 2B: The x-axis label is cut off*

Fixed. *What does it mean that the dominant variability is stimulus driven? It would be nice to speculate about this. You do ask whether they pool disparities around the center but for a larger area and reject that idea.*

To be precise, we do not reject this idea. We just don’t have evidence to suggest that one fixed-size integration area provides a better account of the data than another.

*But what if they use the largest far disparity? The largest near disparity? A randomly chosen small area on the stimulus? Some other strategy that gets knocked around by stimulus details and thus shows variability across stimuli? In other words, what does it mean that different stimuli produce different performance?*

Reviewer 3 had similar questions. We’ve added a new discussion subsection that discusses the results of our simple integration model and speculates on what mechanisms may actually be driving performance on a stimulus-by-stimulus bases (see Stimulus-driven mechanims in the revision). We’ve also tested a few other simple models---namely strategies that use the largest far disparity, largest near disparity, and maximally deviant disparity---which are now presented in the supplement and discussed as well. None of these models fair better than the spatial-integration models.

*Maybe the internal noise variance on a given trial depends, somehow, on the stimulus? You assume additive noise that is fixed, so what if it's Weberian based on something about the stimulus or otherwise multiplicative?*

The analysis assumes that the noise is additive (D=V+W) but---clearly, from the data plots---it does not assume that the variance of the noise is constant. As you are undoubtedly aware, the current methods are blind to trial-by-trial changes in variance. They are sensitive to (i.e. can identify statistical signatures of) trial-by-trial errors (i.e. stimulus specific biases).

The data and analyses clearly show that the noise variance increases log-linearly in both experiments as a function of standard disparity and standard disparity with disparity contrast respectively. A natural way to understand this is that the variance of the internal noise grows with the variance of the stimulus drive.

*Reviewer #2: This manuscript introduces a method for partitioning the variability in a psychophysical task into stimulus-driven components (e.g., disparity and luminance contrast) and internal noise. The primary result, which is consistent with image-computable ideal observer analyses in past work by the same lab on different tasks, is that luminance variability drives perceptual variability substantially. Here, they find that disparity and luminance both contribute to perceptual performance and their contribution is correlated across observers. I think this type of variance decomposition is central to the scientific endeavor when we have “noisy” measurements or “noisy” behavior and the “Law of Total Variance” is a severely underappreciated conceptual framework in perceptual science. The challenge here is partitioning the variance with binary observations governed by truncated cumulative normal distributions. As far as I can tell, the authors got it right and the results are elegant. I have only a few comments.*

Thank you for these remarks.

*My only major comment is that I wonder how different the results would be if you just put together a big logistic regression to predict every choice from the different measurable stimulus components and plot the Beta coefficients. It seems like the main advantage of portioning the variability as the authors did is that it naturally communicates the results in terms of effect size (i.e., what fraction of variance is due to what). I was surprised then that this quantity is reported in terms of “threshold contribution” with no units. My understanding is that these are capturing the variances? Why not represent in the “fraction of total variance”?*

The y axes of Figs 5 and 6 should have units of arcmin---this has been updated in the manuscript.

Threshold contribution T can be written in terms of variance according to equation 6 (T^2=\sigma^2) and can thus be written in terms of fraction of total variance: T^2/T_{total}^2, which is equivalent to decision variable correlation \rho, which we report in the first experiment (Fig 4). We chose to represent the data as threshold contribution because it allows for more direct comparison between the performance of different observers and/or conditions (see text starting at line 240 and new text added to Fig 3 caption), in addition to providing a clear psychophysical interpretation. Also see Reviewer 1’s comment and answer above*.*

Regarding logistic/probit regression… As we understand, a single mixed effects model should produce a similar pattern of results. The challenge would be to properly handle the repreated measures aspect of this experiment. As the reviewers sussed out, we for the current analytical approach because our it allows for results to be couched in signal-detection-theoretic terms.

*Optional clarity suggestions: it would be quite useful to see all the analytic solutions in one place (e.g., a table). I think the field would find this paper more useful if they could easily map the variance partitioning multi-pass experiment framework into their variables of interest. Right now, the presentation is thorough, but there’s a lot of jumping back and forth between equations to follow the logic.*

We’ve added a new figure (Fig 13 in the revision) to the discussion section to help summarize the key findings of this study.

*Typos:

Equation 4: shouldn’t it be the standard deviation instead of the variance? For the formula in equation 3 to be correct, the denominator of d’ should be the common standard deviation. I believe this is probably a typo.*

Fixed. Please see above.

*Section 4.9.3 : Depthnzz*

Fixed.

*Discussion “see Resutls”*

Fixed.

*Reviewer #3: This study applies an elegant logic to the task of separating out different components of variability that affect participants in a stereoacuity task using stereo photographs of natural stimuli. My main comments concern the clarity with which the authors describe the psychophysical task and stimuli. In an ideal world, they should be able to present a figure where the reader can see one of the main effects for themselves.*

This main concern was shared by reviewer #2. We’ve added a figure (Fig 2 in the revision), see below for details. We think this much improves the manuscript. Thank you for the suggestion.

*Major

1. Stimulus and task. It would help the reader to be clear what the stimulus was. p10 the Methods say 'In one interval... standard...In the other interval, ....comparison stimulus..' The assumption is that the same scene is presented in both intervals, with only the pedestal disparity changing. It would be helpful to make this explicit.*

There has been some misunderstanding. The standard and comparison stimuli were drawn from different scenes. That is, the pattern of standard and comparison image patterns were NOT the same in both intervals.

In the “Procedure” subsection we specified that “a unique natural stereo-image patch was presented on each interval of each trial.” The only time a stimulus was viewed twice was in the second pass.

We have added a sentence to the text in the referenced passage to make this point clear at this stage of the manuscript. It reads:

“All stimuli (standards and comparisons) were always unique across all intervals and trials of an experiment, having been sampled from different locations and scenes across the source image set.”

The caption of the new figure we mentioned above (Fig 2 in the revision) reiterates this:

“Each natural image patch was unique across all trials and intervals.”

*The image in Fig 2A is too small to be helpful.*

We agree. We have reformatted the figure (now Fig 3A in the revision) to make better use of the available real estate. We think this has markedly improved the figure.

*Also, for Fig 2A, delta is given as -7.50 arcmin. It might be helpful to change delta_{cmp} to something different to help the reader understand the stimulus.*

Here, we think it would be best to keep the standard and comparison disparities the same because it makes for the most intuitive example to think through how decision variable correlation can be estimated from data, and because it is such trials that provide the most power for estimating decision variable correlation. We have added text to the caption to make this point clear:

“Data at 0.5 proportion comparison chosen (*arrow*) is the least informative for estimating discrimination thresholds, but the most informative for estimating decision variable correlation (see *D* and *E*)”

*Even more helpful would be for the reader to see two examples of a trial, so that they could see whether they get the main effect themselves. For example, the authors could repeat Fig 1C with a different pedestal disparity, then repeat both rows but now with a low disparity range. This should be considerably easier (Fig 4A). The icing on the cake would be a cross section of the stimulus in each case (x-axis showing pixel number across the image, y-axis showing disparity), times four (standard and comparison for high and low disparity contrast) so that readers instantly understands the stimulus and task. At the moment, a lot of reading of text is required to piece together the stimulus and task.*

Good suggestion. We have added a new figure (Fig 2 in the revision). It depicts a single trial from each of the two disparity contrast conditions. The top row shows a trial with image patches having high disparity contrast. The bottom row shows a trial with image patches having low disparity contrast. (Note that the image patterns are unique on each interval of each trial.)

*2. Monocular cues. If the same scene is used for the standard and comparison stimulus then, in theory, the task could be done monocularly (unless the authors sampled the scene with a slightly different lateral position of the scene relative to the camera to avoid this, but I did not find this in the text). This does not affect the authors' analysis (it would just contribute to lowering sigma_L, the luminance-driven variability), but it would be helpful if the authors could comment on this in the text.*

Please see our response to the reviewer comment “1.” above. Given the trial design, there is no way that monocular cues of the sort that the reviewer refers to could be useful for the task. Specifically, because the same scene/image patch was not used on both intervals of a single trial, lateral shifts of an image feature in one (e.g. the left) eye’s image across intervals could not be used to game out what the disparity difference between intervals was. Does that make sense?

*3. Disparity range. I found it hard to pick what the authors' model was for the effect of disparity contrast (although they isolate the effect very clearly). For example, Harris and Parker (1995) discussed the efficiency of integrating disparity information across a number of samples in order to compare the mean disparity of dots spanning a range of disparities. Do the authors envisage a similar integration process? The authors point out that the participant could use the central pixel and perform in the same way as if the disparity contrast was zero, in which case no integration would be necessary.*

Our aim was to characterize the effect of disparity contrast on performance. However, we now briefly discuss several new stimulus-driven mechanistic models in addition to the spatial integration models that we examined in the original submission. None of these models provide an improved account of the trial-by-trial response variability (see paragraphs at line 533, new sentences starting at line 701, and Fig S1).

That said, we’ve taken your comments and those of Review 1 to heart and added a new discussion subsection (see “Stimulus-driven mechanisms” in the revision) and supplemental figure (Fig S1) on these points.

*4. A different experiment. It might be helpful for the reader if the authors mentioned in the Discussion what they thought would happen if the experiment had used an entirely different stereo photograph for the comparison stimulus.This would make the stimulus more similar to other experiments using dynamic random dot patterns where the luminance component is changed but the disparity profile remains. It would still be possible to do double pass experiments, provided the stimulus and comparison were repeated exactly on the second pass. As I understand it, the results of that experiment are not necessarily predictable from the sources of variability that the authors have gathered in the current experiment. Discussing this case would help to clarify how abstract a representation of disparity the authors believe participants retain between the standard and comparison (e.g., if just a single estimate of the pedestal value is retained, then changing between different stereo photographs might not matter).*

This is the experiment that we actually did. Apologies for the confusion! We hope that the additional figure and added text (see above) will prevent similar future misunderstandings.

*Minor

p8. 'high' 'low' disparities, wrong way round.*

Fixed.

*p10. 'that proportion comparison...' Difficult sentence that does not effectively explain the basis on which the stimuli were chosen.*

We’ve cleaned up the sentence to make it easier to understand. It now reads:

“The disparity-contrast levels and the comparison disparities in each condition were chosen based on pilot data. Comparison disparities were chosen with the aim that so that data points on the psychometric function ranged from 10% to 90% in the low disparity contrast condition. Data points at 0% and 100% provide no useful information for estimating decision variable correlation (see subsection "Partitioning the variability of the decision variable" below). Before collecting the data, each observer completed practice sessions to ensure that discrimination performance was stable.”

With respect to stimulus section, the criteria by which individual stimuli were chosen were discussed in the first two paragraphs of the “Stimulus sampling” section.

*Fig 3. Explain the dashed line in the legend.*

Fixed.

*Fig 11. 'affect of' -> effect of*

Fixed.
